# Clinical Outcomes Associated With Overestimation of Oxygen Saturation by Pulse Oximetry in Patients Hospitalized With COVID-19

**DOI:** 10.1001/jamanetworkopen.2023.30856

**Published:** 2023-08-24

**Authors:** Ashraf Fawzy, Tianshi David Wu, Kunbo Wang, Kenneth E. Sands, Arielle M. Fisher, Shanna A. Arnold Egloff, Jeffrey D. DellaVolpe, Theodore J. Iwashyna, Yanxun Xu, Brian T. Garibaldi

**Affiliations:** 1 Division of Pulmonary and Critical Care Medicine, Johns Hopkins University School of Medicine, Baltimore, Maryland; 2 Section of Pulmonary, Critical Care, and Sleep Medicine, Baylor College of Medicine, Houston, Texas; 3 Center for Innovations in Quality, Effectiveness, and Safety, Michael E. DeBakey Veterans Administration Medical Center, Houston, Texas; 4 Department of Applied Mathematics and Statistics, Johns Hopkins University, Baltimore, Maryland; 5 HCA Healthcare, HCA Healthcare Research Institute (HRI), Nashville, Tennessee; 6 HCA Healthcare, Sarah Cannon, Genospace, Nashville, Tennessee; 7 Department of Health Policy and Management, Bloomberg School of Public Health, Johns Hopkins University, Baltimore, Maryland

## Abstract

**Importance:**

Many pulse oximeters have been shown to overestimate oxygen saturation in persons of color, and this phenomenon has potential clinical implications. The relationship between overestimation of oxygen saturation with timing of COVID-19 medication delivery and clinical outcomes remains unknown.

**Objective:**

To investigate the association between overestimation of oxygen saturation by pulse oximetry and delay in administration of COVID-19 therapy, hospital length of stay, risk of hospital readmission, and in-hospital mortality.

**Design, Setting, and Participants:**

This cohort study included patients hospitalized for COVID-19 at 186 acute care facilities in the US with at least 1 functional arterial oxygen saturation (SaO_2_) measurement between March 2020 and October 2021. A subset of patients were admitted after July 1, 2020, without immediate need for COVID-19 therapy based on pulse oximeter saturation (SpO_2_ levels of 94% or higher without supplemental oxygen).

**Exposures:**

Self-reported race and ethnicity, difference between concurrent SaO_2_ and pulse oximeter saturation (SpO_2_) within 10 minutes, and initially unrecognized need for COVID-19 therapy (first SaO_2_ reading below 94% despite SpO_2_ levels of 94% or above).

**Main Outcome and Measures:**

The association of race and ethnicity with degree of pulse oximeter measurement error (SpO_2_ − SaO_2_) and odds of unrecognized need for COVID-19 therapy were determined using linear mixed-effects models. Associations of initially unrecognized need for treatment with time to receipt of therapy (remdesivir or dexamethasone), in-hospital mortality, 30-day hospital readmission, and length of stay were evaluated using mixed-effects models. All models accounted for demographics, clinical characteristics, and hospital site. Effect modification by race and ethnicity was evaluated using interaction terms.

**Results:**

Among 24 504 patients with concurrent SpO_2_ and SaO_2_ measurements (mean [SD] age, 63.9 [15.8] years; 10 263 female [41.9%]; 3922 Black [16.0%], 7895 Hispanic [32.2%], 2554 Asian, Native American or Alaskan Native, Hawaiian or Pacific Islander, or another race or ethnicity [10.4%], and 10 133 White [41.4%]), pulse oximetry overestimated SaO_2_ for Black (adjusted mean difference, 0.93 [95% CI, 0.74-1.12] percentage points), Hispanic (0.49 [95% CI, 0.34-0.63] percentage points), and other (0.53 [95% CI, 0.35-0.72] percentage points) patients compared with White patients. In a subset of 8635 patients with a concurrent SpO_2_ − SaO_2_ pair without immediate need for COVID-19 therapy, Black patients were significantly more likely to have pulse oximetry values that masked an indication for COVID-19 therapy compared with White patients (adjusted odds ratio [aOR], 1.65; 95% CI, 1.33-2.03). Patients with an unrecognized need for COVID-19 therapy were 10% less likely to receive COVID-19 therapy (adjusted hazard ratio, 0.90; 95% CI, 0.83-0.97) and higher odds of readmission (aOR, 2.41; 95% CI, 1.39-4.18) regardless of race (*P* for interaction = .45 and *P* = .14, respectively). There was no association of unrecognized need for COVID-19 therapy with in-hospital mortality (aOR, 0.84; 95% CI, 0.71-1.01) or length of stay (mean difference, −1.4 days; 95% CI, −3.1 to 0.2 days).

**Conclusions and Relevance:**

In this cohort study, overestimation of oxygen saturation by pulse oximetry led to delayed delivery of COVID-19 therapy and higher probability of readmission regardless of race. Black patients were more likely to have unrecognized need for therapy with potential implications for population-level health disparities.

## Key Points

**Question:**

Is overestimation of oxygen saturation by pulse oximeters associated with delayed delivery of COVID-19 specific therapies, hospital readmission, length of stay, or in-hospital mortality, and are associations different by race and ethnicity?

**Findings:**

In this cohort study of 24 504 patients with concurrently measured pulse oximetry and arterial oxygen saturation, pulse oximeters more commonly overestimated arterial oxygen saturation in patients from minority racial and ethnic groups and led to delayed recognition of need for COVID-19 therapy among Black patients compared with White patients. In a subset of 8635 patients without immediate need for COVID-19 therapy on admission, overestimation of oxygen saturation by pulse oximetry was associated with delayed delivery of COVID-19 therapy and increased risk of hospital readmission, irrespective of patient race.

**Meaning:**

These results suggest that although racial and ethnic disparities exist in measurement of oxygen saturation by pulse oximetry, overestimation may increase the risk of hospital readmission regardless of patient race.

## Teaser

This cohort study of US adults treated at acute care facilities for COVID-19 examines the association of overestimation of oxygen saturation by pulse oximetry and clinical outcomes stratified by race and ethnicity.

## Introduction

Pulse oximetry noninvasively estimates arterial oxygen saturation. First developed in the 1970s, it has become ubiquitous in health care settings and influences clinical decision-making.^1^ During the COVID-19 pandemic, oxygen saturation thresholds were used to define disease severity, making the pulse oximeter particularly important for triage and treatment decisions.^2,3^

Observational data and laboratory studies suggest that pulse oximeters systematically overestimate arterial oxygen saturation among patients from minority racial and ethnic groups, leading to a greater risk of occult hypoxemia, generally defined as a true arterial oxygen saturation (SaO_2_) below 88% with an oxygen saturation by pulse oximetry (SpO_2_) in a normal range above 92%.^4,5,6,7,8,9,10^ The presence of occult hypoxemia has been associated with lower rates of supplemental oxygen among critically ill patients.^11^ Our group has shown that pulse oximetry overestimates arterial oxygen saturation and was associated with a higher likelihood of occult hypoxemia in patients from minority racial and ethnic groups admitted to 5 hospitals with COVID-19.^10^ Among Black and Hispanic patients, this overestimation led to a delay in recognizing eligibility for COVID-19 treatments that rely on oxygenation targets.^12^ However, because our previous study relied on statistical modeling to predict arterial oxygen saturation, it remains unknown whether true overestimation of oxygen saturation by pulse oximeters led to a delay in administration of COVID-19 therapies and if such a delay differed based on patients’ race or ethnicity. Furthermore, the health consequences of pulse oximeter inaccuracy among hospitalized patients with COVID-19 have not been examined.

The aim of this study is to determine the association between overestimation of oxygen saturation with delay in administration of COVID-19 therapy, hospital length of stay, risk of hospital readmission, and in-hospital mortality using data from a large national hospital network with specific attention to potential racial and ethnic differences in pulse oximeter performance.

## Methods

### Study Design and Participants

Data was extracted from the COVID-19 Consortium of HCA Healthcare and Academia for Research Generation (CHARGE),^13^ comprising electronic health record data from all patients hospitalized for COVID-19 between March 1, 2020, and October 31, 2021, in 186 HCA Healthcare acute-care facilities in the US. Patients were included based on detection of SARS-CoV-2 by nucleic acid test and specific *International Statistical Classification of Diseases and Related Health Problems, Tenth Revision *(*ICD-10*) codes indicating symptomatic infection, as described elsewhere.^14,15^ We examined the patients within CHARGE who had at least 1 functional arterial oxygen saturation (SaO_2_) measured during at least 1 hospitalization. Logical Observation Identifiers Names and Codes (LOINC) descriptors were used to identify measured functional SaO_2_ for inclusion, and excluded fractional SaO_2_ measures. The study followed the Strengthening the Reporting of Observational Studies in Epidemiology (STROBE) reporting guideline and received a waiver of consent and was deemed minimal risk by both the Johns Hopkins and an external institutional review board (WIRB-Copernicus Group).

### Exposure and Outcome Definitions

For each SaO_2_ value, we identified the nearest estimated oxygen saturation by pulse oximetry (SpO_2_) within 10 minutes of arterial blood sample acquisition. The degree of error in the estimation of oxygen saturation by pulse oximetry was calculated as the difference between SpO_2_ and SaO_2_. Occult hypoxemia was defined as SaO_2_ levels below 88% with concurrent SpO_2_ between 92% and 96%.^4^ Patients were considered to have had an unrecognized need for treatment if the first SaO_2_ in the hospitalization was below 94% despite a 10-minute SpO_2_ of 94% or higher, based on HCA Healthcare System criteria for eligibility for remdesivir and dexamethasone and consistent with recommendations of the Infectious Diseases Society of America and the US Centers for Disease Control.^2,3,16^ A recognized need for treatment was defined as both a SaO_2_ and SpO_2_ below 94%.

Self-reported race and ethnicity were collected from the electronic medical record. Based on an empirical examination of the prevalence of races and ethnicities and consistent with the work of others,^8,11,19,29^ patients were categorized as non-Hispanic White, Black, Hispanic, or other race and ethnicity (Asian, Native American or Alaskan Native, Hawaiian or Pacific Islander, other, and unknown).

We performed 2 separate analyses. In the first analysis, we sought to replicate prior findings by determining the association of race and ethnicity with likelihood of errors in pulse oximetry. For this analysis, race and ethnicity was the primary exposure, and the primary outcomes estimated separately were degree of measurement error (SpO_2_ − SaO_2_), the odds of occult hypoxemia, and odds of unrecognized need for treatment.

In the second analysis, we sought to determine the association of unrecognized need for treatment with health outcomes. This analysis was limited to patients admitted after July 1, 2020, when evidence for the efficacy of both remdesivir and dexamethasone had been reported.^17,18^ The primary exposure was the presence of an unrecognized vs recognized need for treatment based on the first concurrent SpO_2_ and SaO_2_. The primary outcome was time to remdesivir or dexamethasone. In cases where an individual had more than 1 admission, we considered the first. The secondary outcomes included in-hospital mortality (defined as death or discharge to hospice), length of stay, and 30-day readmission, defined as a return to the hospital for any indication within 30 days of discharge.

### Statistical Analysis

The association of race and ethnicity with error in pulse oximetry was examined using a linear mixed effects model with demographic characteristics (age and sex), baseline smoking status (current smoker vs former or never smoker), Charlson Comorbidity Index (CCI), history of diabetes, maximum illness severity defined by World Health Organization (WHO) criteria, time-varying (within hospital stay) vital signs (mean arterial pressure and temperature), and time-varying laboratory results (creatinine, hemoglobin, and total bilirubin) as fixed effects covariates.^12^ Smoking status was based on self-report while CCI and history of diabetes were defined based on *ICD-10* codes. These covariates were selected as they may affect the accuracy of the pulse oximeter. Vital signs were included if they were collected 4 hours before or 2 hours after the SaO_2_ measurement while laboratory results were included if they were collected in the 48 hours preceding SaO_2_ measurement. All covariates and their interactions with race and ethnicity were included as fixed effects. Clustering due to repeated measures within-patient was accounted for using a random intercept and hospital site. Time-varying vital signs and laboratory measurements were additionally included as random effects. An unstructured variance-covariance structure was employed for each random effect and separate random effects were considered to be independent. Only the first hospital stay and records without missing covariate data were included in the adjusted linear mixed-effects model.

The occurrence of occult hypoxemia was presented as the proportion of individuals with at least one instance of occult hypoxemia during their stay in hospital and as the proportion of total SpO_2_ − SaO_2_ measurement pairs consistent with occult hypoxemia by race and ethnicity. A mixed effects logistic regression model adjusted for age, sex, maximum WHO criteria, body mass index (BMI) at admission, current smoking status, and CCI as fixed effects and hospital site as random effects was used to investigate the association between race and ethnicity and unrecognized need for COVID-19 therapy defined as first SaO_2_ measurement below 94% despite SpO_2_ levels of 94% or above. This analysis was limited to patients hospitalized for COVID-19 after July 1, 2020, with admission SpO_2_ 94% or higher without supplement oxygen.

Associations of unrecognized need for treatment with in-hospital mortality and hospital readmission were examined using mixed effects logistic regression models, while the association with time to receipt of COVID-19 therapy was examined using a mixed effects Cox proportional hazard model, and the associations with length of hospital stay were examined using linear mixed effects models. All models were adjusted for race, age, sex, BMI, and CCI as fixed effects and hospital site as a random effect. Models for more remote clinical outcomes (readmission, length of stay, and in-hospital mortality) were additionally adjusted for maximum WHO criteria. Interaction between unrecognized need for treatment with race and ethnicity was tested. Two sensitivity analyses were performed for the evaluation of the association between unrecognized need for treatment with time to receipt of COVID-19 therapy: (1) limiting the sample to SpO_2_ − SaO_2_ pairs in the first day of hospitalization and COVID-19 therapy delivered within the first 96 hours and (2) adding concurrent vital signs (mean arterial pressure and temperature). All hypothesis tests were 2-sided and results were considered statistically significant at *P* < .05. All statistical analyses were conducted using R version 4.0.2 (R Foundation for Statistical Computing), and all mixed effects models were fitted using the linear mixed-effects models (lme) 4 package.

## Results

There were 40 738 patients (mean [SD] age, 63.8 [16.5] years; 17 772 female [43.6%], 7142 Black [17.5%], 12 903 Hispanic [31.7%], 3837 with another race or ethnicity [9.4%], and 16 856 [41.4%] White) with at least 1 SaO_2_ measurement across 186 hospitals, of whom 24 504 patients (10 263 female [41.9%]; 3922 Black [16.0%], 7895 Hispanic [32.2%], 2554 with another race or ethnicity [10.4%], and 10 133 White [41.4%]) had 213 229 SaO_2_ records paired to a SpO_2_ result within 10 minutes (eTable 1 in Supplement 1). Patients from minority racial and ethnic groups were on average younger than White patients. Black patients had the highest proportion of female patients and patients with obesity, diabetes, and 5 or more comorbidities in the CCI, as well as the shortest time to first SaO_2_ measurement (eTable 2 in Supplement 1).

### Association of Race and Ethnicity With Pulse Oximeter Error

**Figure 1.**
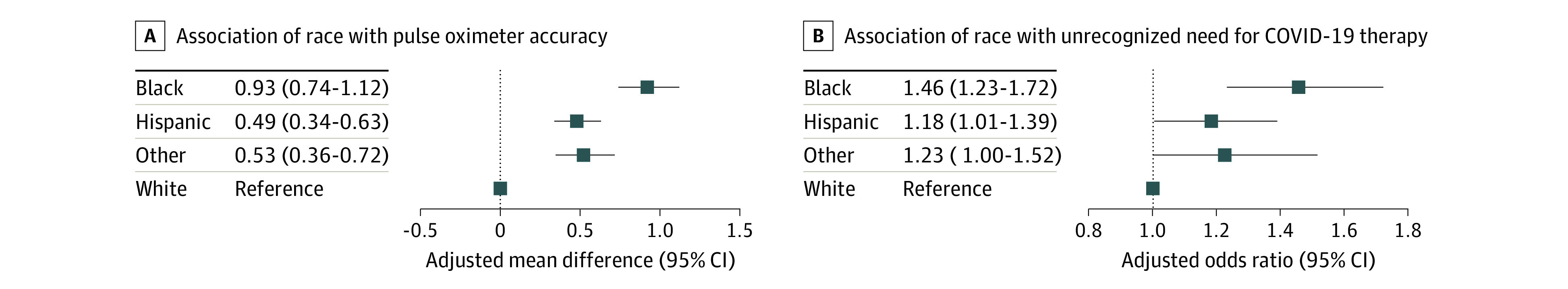
Association of Race and Ethnicity With Pulse Oximeter Accuracy and Delayed Recognition of Need for COVID-19 Therapy Other racial and ethnic minority patients included Asian, Native American or Alaskan Native, Hawaiian or Pacific Islander, and patients with another race or ethnicity. A. Pulse oximeter accuracy defined as mean difference between pulse oximeter saturation (SpO_2_) and arterial oxygen saturation (SaO_2_). B. Delayed recognition defined as first SaO_2_ measurement below 94% despite SpO_2_ of 94% or above.

Among Black and Hispanic patients, SpO_2_ consistently overestimated SaO_2_ at each reading (eFigure in Supplement 1). Occult hypoxemia (ie, SaO_2_ below 88% despite SpO_2_ levels between 92% and 96%) occurred in 719 of 3922 Black patients (18.3%), 1649 of 7895 Hispanic patients (20.9%), and 502 of 2554 patients from other racial and ethnic minority groups (19.7%) compared with 1322 of 10 133 White patients (13.0%). At the individual measurement level, occult hypoxemia was noted in 1517 of 31 116 SpO_2_ − SaO_2_ pairs (4.9%) among Black patients, 4157 of 81 728 (5.1%) among Hispanic patients, and 1244 of 27 879 (4.5%) among other racial ethnic minority patients compared with 2529 of 72 506 (3.5%) among White patients. After adjusting for covariates, SpO_2_ significantly overestimated SaO_2_ by 0.93 (95% CI, 0.74-1.12) percentage points among Black patients, by 0.49 (95% CI, 0.34-0.63) percentage points among Hispanic patients, and by 0.53 (95% CI, 0.36-0.72) percentage points among patients from other racial or ethnic minority groups compared with White patients (Figure 1; eTable 3 in the Supplement).

### Association of Race and Ethnicity With Unrecognized Eligibility for COVID-19 Therapy

When considering oxygen saturation criteria for initiation of COVID-19 therapy, there were 15 960 (39.2%) eligible patients with a visit date after July 1, 2020, and admission SpO_2_ of 94% or higher in the absence of supplemental oxygen, of whom 8635 (54.1%) had at least 1 concurrent SpO_2_ − SaO_2_ pair and were included in this analysis. Patients from minority racial and ethnic groups were on average younger than White patients (eg, mean [SD] age: Black, 62.1 [16.5] years vs 68.9 [15.1] years), while a higher proportion of Black patients were female (842 of 1569 [53.7%] vs 1667 female White patients [43.5%]) and had BMI 30 or above (670 [42.7%] vs 1485 [38.8%]). Hispanic patients and those with other race and ethnicity had the fewest comorbidities (CCI score 5 or higher: 829 of 2349 patients [35.3%] vs 1425 White patients [37.2%]), although Hispanic patients had the highest prevalence of diabetes (1472 patients [62.7%] vs 1886 of 3832 White patients [49.2%]) (Table 1). The overall demographics of this subgroup did not differ substantially compared with the parent cohorts (eTable 1 in Supplement 1). Notably, Black patients had the lowest in-hospital mortality and shortest length of stay. In the mixed effects logistic regression model, compared with White patients, Black and Hispanic patients were significantly more likely to have unrecognized need for COVID-19 therapy by oxygen saturation (defined as SpO_2_ of 94% or higher despite having SaO_2_ levels below 94%). The effect size was higher among Black patients (adjusted odds ratio [aOR], 1.46; 95% CI, 1.23-1.72) than Hispanic patients (aOR, 1.18; 95% CI, 1.01-1.39) but not significant among patients of other race and ethnicity (aOR, 1.23; 95% CI, 1.00-1.52) (Figure 1).

**Table 1.** Baseline Characteristics of Cohort^a^

Characteristic	Patients, No. (%) (N = 8635)			
	White, non-Hispanic (n = 3832)	Black (n = 1569)	Hispanic (n = 2349)	Other (n = 885)^b^
Age, mean (SD), y	68.9 (15.1)	62.1 (16.5)	61.5 (17.2)	63.4 (16.8)
Sex				
Female	1667 (43.5)	842 (53.7)	1027 (43.7)	339 (38.3)
Male	2165 (56.5)	727 (46.3)	1322 (56.3)	546 (61.7)
BMI				
<18.5	91 (2.4)	32 (2.0)	25 (1.1)	26 (2.9)
18.5 to <30	1688 (44.1)	594 (37.9)	1016 (43.3)	459 (51.9)
≥ 30	1485 (38.8)	670 (42.7)	961 (40.9)	290 (32.8)
CCI				
0	457 (11.9)	230 (14.7)	387 (16.5)	126 (14.2)
1-4	1950 (50.9)	685 (43.7)	1133 (48.2)	474 (53.6)
≥5	1425 (37.2)	654 (41.7)	829 (35.3)	285 (32.2)
Current smoker	267 (7.0)	102 (6.5)	91 (3.9)	41 (4.6)
Diabetes	1886 (49.2)	934 (59.5)	1472 (62.7)	499 (56.4)
COPD	1494 (39.0)	463 (29.5)	489 (20.8)	178 (20.1)
PVD	653 (17.0)	210 (13.4)	223 (9.5)	92 (10.4)
CKD	1391 (36.3)	672 (42.8)	817 (34.8)	306 (34.6)
WHO				
2	628 (16.4)	400 (25.5)	484 (20.6)	113 (12.8)
3	1618 (42.2)	622 (39.6)	900 (38.3)	281 (31.8)
4	398 (10.4)	129 (8.2)	293 (12.5)	110 (12.4)
5	593 (15.5)	186 (11.9)	303 (12.9)	130 (14.7)
6	595 (15.5)	232 (14.8)	369 (15.7)	251 (28.4)
SpO_2_, mean (SD), %	94.5 (6.2)	94.9 (7.3)	94.6 (5.8)	94.8 (5.6)
Temperature, mean (SD), °C	37.3 (0.8)	37.3 (0.7)	37.3 (0.8)	37.4 (0.8)
Mean (SD) arterial pressure, mm Hg	87.5 (17.2)	89.3 (18.5)	86.9 (20.6)	87.2 (17.2)
Creatinine, mg/dL	1.6 (1.5)	2.6 (3.0)	1.8 (2.1)	1.8 (2.1)
Hemoglobin, g/dL	11.9 (2.5)	11.4 (2.6)	11.9 (2.6)	11.8 (2.6)
Total bilirubin, mg/dL	0.79 (1.28)	0.73 (1.05)	0.86 (1.58)	0.83 (1.06)
Time to first SaO_2_ measurement, median (IQR), h	7.4 (1.2-78.2)	6.2 (1.1-70.3)	11.8 (1.7-76.6)	6.9 (1.3-63.7)
Length of stay, median (IQR), d	11.2 (5.6-18.4)	11.6 (5.3-20.7)	13.4 (6.6-23.3)	12.5 (6.5-21.6)
Died during hospitalization	1828 (47.7)	640 (40.8)	1116 (47.5)	428 (48.4)

Abbreviations: BMI, body mass index (calculated as weight in kilograms divided by height in meters squared); CCI, Charlson Comorbidity Index; CKD, chronic kidney disease; COPD, chronic obstructive pulmonary disease; PVD, peripheral vascular disease; SaO_2_, functional arterial oxygen saturation; SpO_2_, pulse oximeter saturation; WHO, World Health Organization.

To convert bilirubin to micromoles per liter, multiply by 17.104; creatinine to micromoles per liter, multiply by 88.4; hemoglobin to gallons per liter, multiply by 10.0; mean arterial pressure to kilopascals, multiply by 0.133.

^a^ Includes patients with COVID-19, 1 or more paired SpO_2_ − SaO_2_ measurements, and admission SpO_2_ levels 94% or higher without supplement oxygen.

^b^ Other combines the following racial categories, including Asian, Native American or Alaskan Native, Hawaiian or Pacific Islander, other, and unknown.

### Association Between Unrecognized Eligibility for COVID-19 Therapy with Receipt of COVID-19 Therapy and Health Outcomes

**Figure 2.**
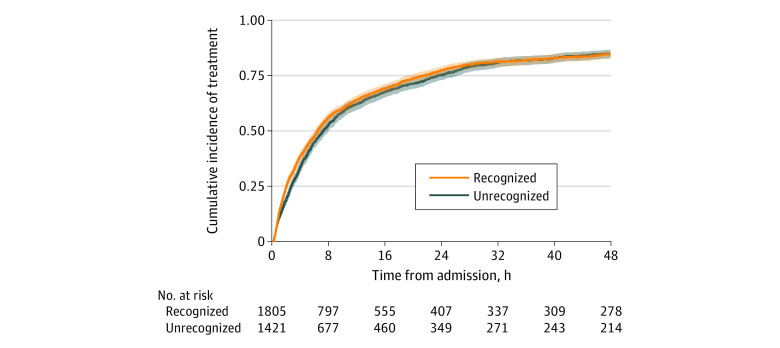
Cumulative Incidence of Treatment During the First 48 Hours of Hospitalization Comparison of patients with COVID-19 admitted after July 1, 2020, with first arterial oxygen saturation (SaO_2_) measurement below 94% who had recognized or unrecognized need for COVID-19 therapy (concurrent pulse oximeter saturation [SpO_2_] at 94% or 94% and higher, respectively).

Patients who experienced an initially unrecognized need for COVID-19 therapy based on pulse oximetry error received treatment at a median (interquartile range [IQR]) of 7.3 hours (2.8-23.4 hours) compared with 6.5 hours (2.0-21.3 hours) for those whose need for therapy was recognized (Figure 2) and had a 10% lower hazard of receiving COVID-19 therapy (adjusted hazard ratio [aHR], 0.90; 95% CI, 0.83-0.97). They also had significantly higher odds of readmission (aOR, 2.41; 95% CI, 1.39-4.18). Among the 273 patients who never received therapy, 3.7% were readmitted compared with 2% of those who eventually received therapy (3226 patients; *P* = .14). There was no effect modification when considering the interaction between unrecognized need for COVID-19 therapy and race for hazard of receiving therapy (*P* for interaction = .45; Table 2) or odds of readmission (*P* for interaction = .14) and race was not significantly associated with either outcome after accounting for unrecognized need for COVID-19 therapy (eTable 4 in Supplement 1). The association between unrecognized need for COVID-19 therapy and delay in receipt of COVID-19 therapy was similar when the sample was limited to SpO_2_ − SaO_2_ pairs in the first day of hospitalization and COVID-19 therapy delivered within the first 96 hours (aHR, 0.88; 95% CI, 0.79-0.98) and when vital signs were added to the multivariable model (aHR, 0.90; 95% CI, 0.83-0.98). Patients with unrecognized need for COVID-19 therapy had point estimates of lower in-hospital mortality (aOR, 0.84; 95% CI, 0.71-1.01) and shorter length of stay (mean difference, −1.4 days; 95% CI, −3.1 to 0.2 days) but neither were statistically significant.

**Table 2.** Median Time to COVID-19 Therapy and Adjusted Hazard Ratios of Timeliness of COVID-19 Therapy Stratified by Race and Ethnicity

Characteristic	Time to therapy, median (IQR), hours		Adjusted hazard ratio (95% CI)	*P* value^a^
	Unrecognized	Recognized		
Overall	7.3 (2.8-23.4)	6.5 (2.0-21.3)	0.90 (0.83-0.98)	.02
Race stratified				
Black	9.5 (3.6-27.6)	7.4 (3.0-24.7)	0.88 (0.72-1.07)	.45
Hispanic	6.4 (2.6-20.8)	5.0 (1.5-16.3)	0.83 (0.71-0.96)	
White	7.1 (2.8-24.8)	7.2 (2.6-23.6)	0.96 (0.85-1.10)	
Other^b^	6.8 (2.3-13.0)	6.1 (2.1-18.4)	1.00 (0.77-1.29)	

^a^ *P* values for stratified race categories are for interaction.

^b^ Other combines the following racial categories, including Asian, Native American or Alaskan Native, Hawaiian or Pacific Islander, other, and unknown.

## Discussion

This retrospective study in a large health care system demonstrated that overestimation of oxygen saturation by pulse oximetry led to delayed delivery of COVID-19 targeted therapy and higher probability of readmission. This analysis expands upon our previous work, which relied on statistical modeling to estimate delays in recognizing need for COVID-19 therapy based on pulse oximeter-associated racial and ethnic differences,^12^ by showing that overestimation of oxygen saturation by pulse oximeters are associated with actual delays in receipt of COVID-19 therapies. Additionally, after accounting for delayed recognition attributable to pulse oximeter inaccuracy, there were no significant racial or ethnic differences in time to receipt of COVID-19 therapy or readmissions, further suggesting that pulse oximeter inaccuracies are central to the racial and ethnic differences in receipt of COVID-19 therapies we previously reported.^12^ Taken together, these findings paint a complementary picture of the impact of pulse oximeter inaccuracy in clinical decision-making and patient outcomes.

Importantly, our analysis investigating the association of pulse oximeter inaccuracy with clinical outcomes only included patients who had an arterial blood gas measurement that led to recognition of severe COVID-19. This is a highly selective population that would be expected to minimize disparities in outcomes. Despite this, there were statistically significant differences in time to drug and readmissions. Individuals for whom pulse oximetry overestimated true oxygen saturation who did not ultimately have an arterial blood gas are not captured by this study and would potentially have poorer outcomes.

We also reconfirmed that pulse oximeters more commonly overestimate oxygen saturation in patients from minority racial and ethnic groups, consistent with several retrospective studies of clinical data in other populations.^4,5,6,11,19^ The magnitude of overestimation of oxygen saturation by pulse oximeters in this study for patients from minority racial and ethnic groups, approximately 0.5 to 1 percentage points higher than White patients, is equivalent to findings of prior studies. Although the absolute difference is modest, this study demonstrates that such small differences are associated with clinical outcomes when oxygen saturation thresholds are used to guide medical decision-making.

We found that hospital readmissions were significantly higher among individuals whose need for COVID-19 treatment was initially unrecognized, although it is unclear whether this is due to delayed administration of treatment or other factors. Randomized clinical trials of remdesivir and dexamethasone in hospitalized patients with COVID-19 that demonstrated efficacy with regards to clinical improvement and mortality did not specifically evaluate readmission as an outcome.^18,20,21,22^ Several observational studies of COVID-19 patient readmission did not consider COVID-19–directed therapies as a potential risk factor.^23,24,25^ However, 1 observational study showed a trend toward fewer readmissions among patients who received remdesivir while 2 studies from Spain identified glucocorticoid use as a risk factor for readmissions.^26,27,28^

The lack of association of delayed recognition of treatment eligibility with mortality and length of stay may be due to the focus on an exposure that is occurring relatively early in the hospital course, the relative clinical impact of different COVID-19 directed therapies, and individual variability of pulse oximeter accuracy throughout the hospital stay.^29^ The trend toward shorter length of stay and lower in-hospital mortality were likewise relatively small and would appear to contradict prior studies.^19^ However, this study’s sample differs substantially with a specific focus on a subgroup of patients who were initially on room air but were eventually recognized to have more severe disease, and we did not have access to out-of-hospital mortality to examine for possible biases from differential censoring by hospital discharge. We also used a higher oxygen saturation threshold in order to investigate clinical decision-making early in the course of hospitalization.

While pulse oximeter inaccuracy was highest among patients from minority racial and ethnic groups, suggesting that these groups would be disproportionately impacted on a population level, approximately 13% of patients who experienced occult hypoxemia and over 43% of patients whose need for COVID-19 therapy went unrecognized by pulse oximetry were White. Variability in the accuracy of pulse oximeters has been previously reported, and the impact it has shown in patients of all races emphasizes the multifactorial nature of pulse oximeter accuracy, extending beyond skin pigmentation.^29,30^ In addition, while this study focused on a relatively homogeneous population of patients diagnosed with COVID-19 to capitalize on the oxygen saturation-based treatment threshold, clinicians rely on pulse oximetry to make triage and therapy decisions in many other acute respiratory illnesses where there would potentially be delays in care due to overestimation of oxygen saturation by pulse oximetry. While these factors might play a greater role for patients from minority racial and ethnic groups due to the higher incidence of inaccuracies, clinicians need to be mindful of the potential for pulse oximetry inaccuracies in all patients in which oxygenation affects treatment decisions and clinical outcomes.

### Limitations

This study had several limitations. Some patients might have been started on oxygen therapy due to dyspnea irrespective of oxygen saturation and thus would have been excluded from this study leading to selection bias. The findings of this study may not be generalizable to the broader population of individuals with COVID-19 as there is confounding by indication for an arterial blood gas inherent in the selection of the study sample. Time to treatment administration may have been affected by several unmeasured variables such as individual practice patterns, availability of therapy, hospital staffing, and census. We also used a single standardized threshold for initiation of therapies based on established guidelines, although certain hospitals or localities may have deviated from these guidelines leading to misclassification of unrecognized need for treatment if a lower threshold was set. By relying on the existence of a clinically measured arterial blood gas to define our patient sample, individual and regional practice patterns for obtaining arterial blood gases and differences in hemoximeter brands and calibration across sites may have introduced sampling bias. Although we intended to exclude fractional saturation measures using LOINC (Logical Observation Identifiers Names and Codes), it is possible that a small number of fractional saturations may have been misclassified or miscoded as functional saturations. As with prior retrospective studies of clinical data, this study relies on self-reported race and ethnicity as a surrogate for skin tone, which does not account for the heterogeneity of skin tones within each racial and ethnic group or regional differences in composition of certain racial and ethnic communities. Furthermore, our data did not have a sufficient sample size of patients from specific racial and ethnic groups other than Black, Hispanic, and White, leading to a consolidated categorization of all other groups. Finally, information on oximeter brands, models, and probe types was not included in the clinical data set and varied within and between sites and over time, therefore we are unable to make any inferences regarding specific pulse oximeters.

## Conclusions

Among patients whose oxygen saturation was overestimated by pulse oximetry, delivery of oxygen threshold–specific COVID-19 therapy was delayed and hospital readmissions occurred more frequently. Improved accuracy of pulse oximeters, which play an important role in triage and treatment decisions, is critical to delivery of timely and equitable care to patients with COVID-19. The implications of pulse oximeter errors likely extend to other acute respiratory illnesses and oxygen supplementation in chronic respiratory disease, which necessitate ongoing investigation.^31^

## Notes

Supplement 1. **eTable 1.** Baseline Characteristics Comparing Individuals Admitted With COVID-19 With at Least One SaO_2_ Measured to the Analytic Samples Used Throughout the Study **eTable 2.** Baseline Characteristics by Race and Ethnicity of Patients Hospitalized in the HCA Healthcare System for COVID-19 With at Least One Concurrent Pair of SaO_2_ and SpO_2_ Measurements Within 10 Minutes **eFigure.** Difference Between Arterial Oxygen Saturation and Pulse Oximetry (SaO_2_ − SpO_2_) by Race and Ethnicity Stratified by SpO_2_ **eTable 3.** Results of the Linear Mixed Model Investigating the Association Between Race and Pulse Oximeter Accuracy **eTable 4.** Results of Linear Mixed Models for the Association of Unrecognized Need for COVID-19 Therapy With Time to COVID-19 Therapy and Readmissions

Supplement 2.
Data Sharing Statement
