# Supplementary material for: Clinical Outcomes Associated With Overestimation of Oxygen Saturation by Pulse Oximetry in Patients Hospitalized With COVID-19
Source: JAMA Netw Open. 2023 Aug 24;6(8):e2330856. doi: 10.1001/jamanetworkopen.2023.30856 (PMC10450566; doi:10.1001/jamanetworkopen.2023.30856)
Supplement: Supplement 1. — eTable 1. Baseline Characteristics Comparing Individuals Admitted With COVID-19 With at Least One SaO2 Measured to the Analytic Samples Used Throughout the Study eTable 2. Baseline Characteristics by Race and Ethnicity of Patients Hospitalized in the HCA Healthcare System for COVID-19 With at Least One Concurrent Pair of SaO2 and SpO2 Measurements Within 10 Minutes eFigure. Difference Between Arterial Oxygen Saturation and Pulse Oximetry (SaO2 − SpO2) by Race and Ethnicity Stratified by SpO2 eTable 3. Results of the Linear Mixed Model Investigating the Association Between Race and Pulse Oximeter Accuracy eTable 4. Results of Linear Mixed Models for the Association of Unrecognized Need for COVID-19 Therapy With Time to COVID-19 Therapy and Readmissions [file jamanetwopen-e2330856-s001.pdf]

Supplemental Online Content

Fawzy A, Wu TD, Wang K, et al. Clinical outcomes associated with overestimation of oxygen saturation by pulse oximetry in patients hospitalized with COVID-19. *JAMA Netw Open.* 2023;6(8):e2330856. doi:10.1001/jamanetworkopen.2023.30856

eTable 1. Baseline Characteristics Comparing Individuals Admitted With COVID-19 With at Least One SaO_2 Measured to the Analytic Samples Used Throughout the Study

eTable 2. Baseline Characteristics by Race and Ethnicity of Patients Hospitalized in the HCA Healthcare System for COVID-19 With at Least One Concurrent Pair of SaO_2 and SpO_2 Measurements Within 10 Minutes

eFigure. Difference Between Arterial Oxygen Saturation and Pulse Oximetry (SaO_2 − SpO_2) by Race and Ethnicity Stratified by SpO_2

eTable 3. Results of the Linear Mixed Model Investigating the Association Between Race and Pulse Oximeter Accuracy

eTable 4. Results of Linear Mixed Models for the Association of Unrecognized Need for COVID-19 Therapy With Time to COVID-19 Therapy and Readmissions

This supplemental material has been provided by the authors to give readers additional information about their work.

© 2023 Fawzy A et al. *JAMA Network Open.*

eTable 1. Baseline characteristics comparing individuals admitted with COVID-19 with at least one SaO_2 measured (N=40,738) to the analytic samples used throughout the study to investigate: (1) the association of race with pulse oximeter accuracy (N=24,504), (2) the association between race and unrecognized need for COVID-19 therapy (N=15,960), and (3) the association between unrecognized need for COVID-19 therapy and clinical outcomes (N= 3,566)

Characteristic [mean ± SD or N (%) unless otherwise specified]	At least one SaO_2 measured (N = 40,738)	At least one concurrent SaO_2 and SpO_2 measurement within ± 10 minutes (N = 24,504)	At least one SaO_2 measured, admission SpO_2 ≥94% without supplement oxygen, admitted after July 1, 2020 (N = 15,960)	At least one concurrent SpO_2-SaO_2 pair, admission SpO_2 ≥94% without supplement oxygen, admitted after July 1, 2020 (N = 8,635)	At least one concurrent SpO_2-SaO_2 pair, first SaO_2 <94%, admitted after July 1, 2020 (N= 3,566)
Age (years)	63.8 (16.5)	63.9 (15.8)	64.9 (17.2)	65.1 (16.5)	65.4 (15.8)
Female	17772 (43.6%)	10263 (41.9%)	7468 (46.8%)	3875 (44.9%)	1598 (44.8%)
Race					
Black	7142 (17.5%)	3922 (16.0%)	3201 (20.1%)	1569 (18.2%)	662 (18.6%)
Hispanic	12903 (31.7%)	7895 (32.2%)	4358 (27.3%)	2349 (27.2%)	1027 (28.8%)
Other	3837 (9.4%)	2554 (10.4%)	1430 (9.0%)	885 (10.2%)	355 (10.0%)
White	16856 (41.4%)	10133 (41.4%)	6971 (43.7%)	3832 (44.4%)	1522 (42.7%)
BMI					
<18.5	668 (1.6%)	382 (1.6%)	334 (2.1%)	174 (2.0%)	54 (1.5%)
18.5 to <30	17165 (42.1%)	9974 (40.7%)	7188 (45.0%)	3757 (43.5%)	1459 (40.9%)
≥ 30	17308 (42.5%)	10907 (44.5%)	6028 (37.8%)	3406 (39.4%)	1566 (43.9%)
CCI					
0	8444 (20.7%)	4616 (18.8%)	2818 (17.7%)	1200 (13.9%)	442 (12.4%)
1-4	21451 (52.7%)	12789 (53.1%)	7935 (49.7%)	4242 (49.1%)	1801 (50.5%)
≥ 5	10843 (26.6%)	7099 (29.5%)	5207 (32.6%)	3193 (37.0%)	1323 (37.1%)
Current Smoker	2173 (5.3%)	1252 (5.1%)	980 (6.1%)	501 (5.8%)	171 (4.8%)

© 2023 Fawzy A et al. *JAMA Network Open.*

Characteristic [mean ± SD or N (%) unless otherwise specified]	At least one SaO_2 measured (N = 40,738)	At least one concurrent SaO_2 and SpO_2 measurement within ± 10 minutes (N = 24,504)	At least one SaO_2 measured, admission SpO_2 ≥94% without supplement oxygen, admitted after July 1, 2020 (N = 15,960)	At least one concurrent SpO_2-SaO_2 pair, admission SpO_2 ≥94% without supplement oxygen, admitted after July 1, 2020 (N = 8,635)	At least one concurrent SpO_2-SaO_2 pair, first SaO_2 <94%, admitted after July 1, 2020 (N= 3,566)
Diabetes	20471 (50.3%)	12927 (52.8%)	8276 (51.9%)	4791 (55.5%)	2081 (58.4%)
COPD	11308 (27.8%)	7137 (29.1%)	4789 (30.0%)	2624 (30.4%)	1046 (29.3%)
PVD	3954 (9.7%)	2870 (11.7%)	2004 (12.6%)	1178 (13.6%)	478 (13.4%)
CKD	11414 (28.0%)	7918 (32.3%)	5240 (32.8%)	3186 (36.9%)	1379 (38.7%)
WHO					
2	6171 (15.1%)	2638 (10.8%)	3948 (24.7%)	1625 (18.8%)	644 (18.1%)
3	18918 (46.4%)	9457 (38.6%)	7340 (46.0%)	3421 (39.6%)	1582 (44.4%)
4	5924 (14.5%)	4174 (17.0%)	1380 (8.6%)	930 (10.8%)	470 (13.2%)
5	5604 (13.8%)	4343 (17.7%)	1693 (10.6%)	1212 (14.0%)	483 (13.5%)
6	4121 (10.1%)	3846 (15.7%)	1599 (10.0%)	1447 (16.8%)	387 (10.9%)
SpO_2 (%)	93.6 (6.5)	93.7 (6.4)	94.7 (6.2)	94.7 (6.2)	92.0 (6.9)
Temperature (°C)	37.4 (0.8)	37.4 (0.8)	37.3 (0.8)	37.3 (0.8)	37.3 (0.8)
Mean Arterial Pressure (mmHg)	87.5 (17.2)	87.5 (17.2)	87.6 (18.5)	87.6 (18.5)	87.9 (19.5)
Creatinine (mg/dL)	1.6 (1.8)	1.6 (1.8)	1.9 (2.1)	1.9 (2.1)	1.8 (2.0)
Hemoglobin (g/dL)	12.3 (2.5)	12.4 (2.5)	11.8 (2.6)	11.8 (2.6)	11.9 (2.5)
Total Bilirubin (mg/dL)	0.77 (1.0)	0.77 (1.0)	0.80 (1.3)	0.80 (1.3)	0.8 (1.5)
Time (hours) to first SaO_2	5.9 (1.2 – 54.7)	6.1 (1.1 – 59.7)	6.9 (1.4 – 65.5)	7.9 (1.3 – 74.3)	19.1 (1.7 – 101.1)
LOS (days; median [IQR])	10.0 (5.2 – 17.7)	13.2 (7.0 – 22.2)	8.6 (4.4 – 16.1)	12.1 (6.0 – 20.4)	13.3 (7.3 – 21.5)
Died during hospitalization	14568 (35.8%)	12267 (50.1%)	5060 (31.7%)	4012 (46.5%)	1927 (54.0%)

© 2023 Fawzy A et al. *JAMA Network Open.*

eTable 2. Baseline characteristics by race/ethnicity of patients hospitalized in the HCA Healthcare system for COVID-19 with at least one concurrent pair of SaO_2 and SpO_2 measurements within ± 10 minutes

**Characteristic [mean ± SD or N (%) unless otherwise specified]**	**White, non-Hispanic (N = 10133)**	**Black (N = 3922)**	**Hispanic (N = 7895)**	**Other (N = 2554)**
Age (years)	67.5 (14.6)	61.5 (15.8)	60.9 (16.3)	62.5 (15.7)
Female	4134 (40.8%)	2009 (51.2%)	3158 (40.0%)	962 (37.7%)
BMI				
<18.5	195 (1.9%)	69 (1.8%)	62 (0.8%)	56 (2.2%)
18.5 to <30	4170 (41.2%)	1427 (36.4%)	3110 (39.4%)	1267 (49.6%)
≥ 30	4431 (43.7%)	1842 (47.0%)	3709 (47.0%)	925 (36.2%)
Charleston Comorbidity Index				
0	1718 (17.0%)	649 (16.5%)	1703 (21.6%)	546 (21.4%)
1-4	5384 (53.9%)	1886 (48.5%)	4141 (53.7%)	1378 (55.3%)
≥ 5	3031 (30.4%)	1387 (35.7%)	2051 (26.6%)	630 (25.3%)
Current Smoker	595 (5.9%)	252 (6.4%)	296 (3.7%)	109 (4.3%)
Diabetes	4728 (46.7%)	2300 (58.6%)	4579 (58.0%)	1320 (51.7%)
COPD	3777 (37.3%)	1119 (28.5%)	1695 (21.5%)	546 (21.4%)
PVD	1491 (14.7%)	410 (10.5%)	732 (9.3%)	237 (9.3%)
CKD	3251 (32.1%)	1529 (39.0%)	2380 (30.1%)	758 (29.7%)
WHO				
2	956 (9.4%)	601 (15.3%)	884 (11.2%)	197 (7.7%)
3	4111 (40.6%)	1609 (41.0%)	2951 (37.4%)	786 (30.8%)
4	1678 (16.6%)	494 (12.6%)	1500 (19.0%)	502 (19.7%)
5	1923 (19.0%)	631 (16.1%)	1343 (17.0%)	446 (17.5%)
6	1445 (14.3%)	578 (14.7%)	1203 (15.2%)	620 (24.3%)
SpO_2 (%)	93.5 (6.4)	94.4 (6.6)	93.5 (6.4)	93.9 (6.4)
Temperature (°C)	37.3 (0.8)	37.3 (0.8)	37.4 (0.8)	37.5 (0.9)
Mean Arterial Pressure (mmHg)	87.4 (16.5)	89.7 (17.6)	87.0 (17.9)	86.7 (16.7)
Creatinine (mg/dL)	1.5 (1.4)	2.2 (2.5)	1.6 (1.8)	1.6 (1.7)
Hemoglobin (g/dL)	12.4 (2.5)	11.8 (2.5)	12.5 (2.5)	12.5 (2.5)
Total Bilirubin (mg/dL)	0.8 (1.1)	0.7 (0.8)	0.8 (1.0)	0.8 (1.2)
Time (hours) to first SaO_2	5.9 (1.0)	4.1 (0.9)	8.0 (1.4)	5.8 (1.2)

‘Other’ combines the following racial categories: Asian, Native American or Alaskan Nat, Hawaiian or Pacific Islander, Other, and Unknown

© 2023 Fawzy A et al. *JAMA Network Open.*

eFigure. Difference between arterial oxygen saturation and pulse oximetry (SaO_2-SpO_2) by race/ethnicity stratified by SpO_2

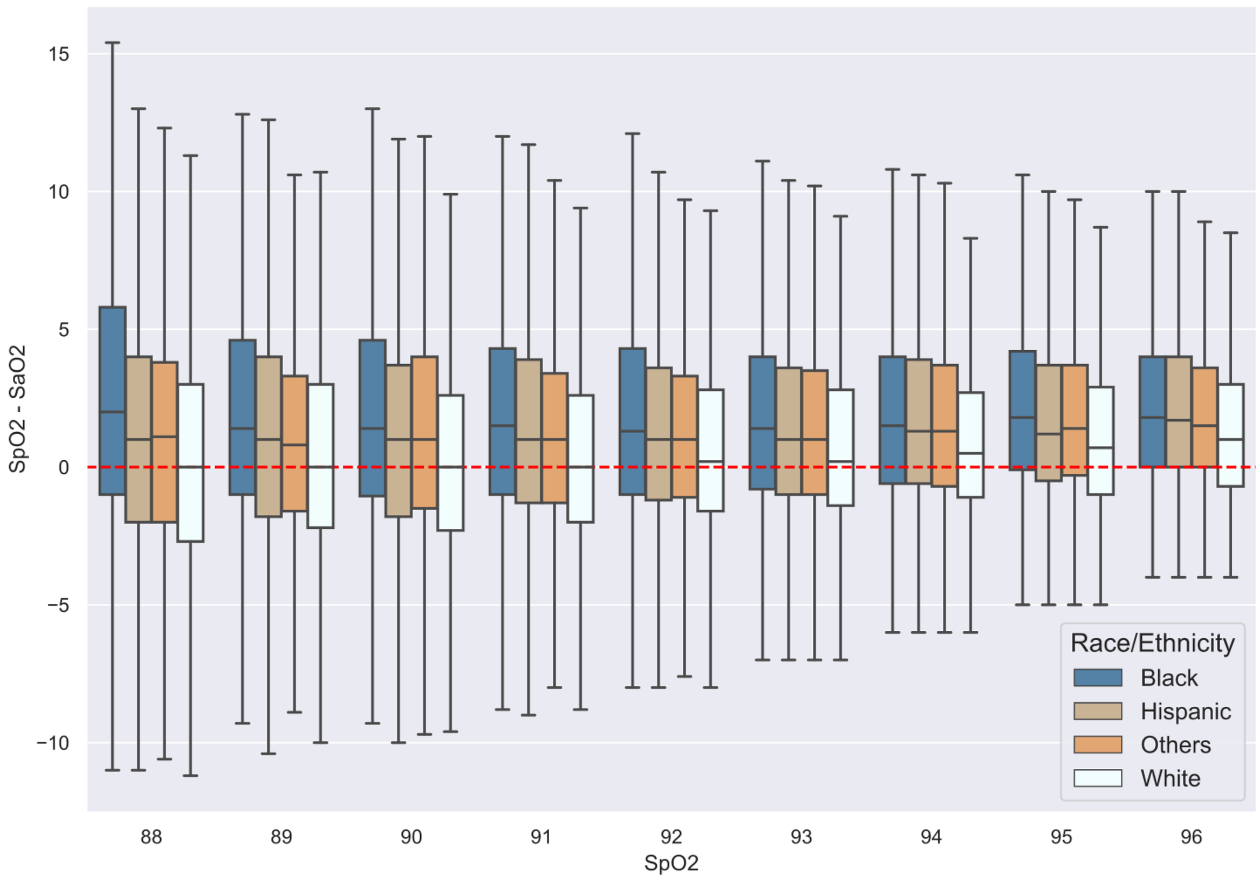

© 2023 Fawzy A et al. *JAMA Network Open.*

eTable 3. Results of the linear mixed model investigating the association between race and pulse oximeter accuracy

Variable	Estimate	Standard Error	p-value
(Intercept)	-1.88662	0.31764	3.03E-09
spo2_s	-1.57537	0.055789	<1E-10
map_s	0.095282	0.035618	0.007487
tempc_s	0.022528	0.030833	0.465039
age	-0.00146	0.004943	0.767047
age_l	0.134988	0.138172	0.328612
race1Black	-0.48005	0.554643	0.386774
race2Hispanic	0.169019	0.398879	0.671765
race3Others	-0.2974	0.575429	0.605277
male	0.155765	0.08732	0.074471
hfnc_or_niv	0.174232	0.141096	0.216892
vent	0.40332	0.117437	0.000594
creatinine_s	-0.22967	0.051652	9.01E-06
bilitot_s	-0.38875	0.095203	5.53E-05
hgb_s	0.346435	0.0401	6.65E-18
diabetes	-0.18157	0.097276	0.061992
cci1	0.160143	0.127512	0.209172
cci2	0.026172	0.15829	0.868677
smoking	-0.22437	0.188732	0.234517
spo2_s:race1Black	-0.06242	0.106279	0.557012
spo2_s:race2Hispanic	0.166891	0.077075	0.030386
spo2_s:race3Others	0.14813	0.11167	0.184704
map_s:race1Black	0.023393	0.066463	0.724872
map_s:race2Hispanic	-0.00031	0.049052	0.994923
map_s:race3Others	0.031002	0.06856	0.651153
tempc_s:race1Black	-0.01953	0.061581	0.751155
tempc_s:race2Hispanic	-0.05199	0.042498	0.221257
tempc_s:race3Others	-0.04608	0.058513	0.431033
age:race1Black	-0.00318	0.009306	0.732412
age:race2Hispanic	0.00143	0.006655	0.8299
age:race3Others	0.004303	0.009416	0.647719
age_l:race1Black	0.017153	0.267881	0.948947
age_l:race2Hispanic	-0.15646	0.1922	0.415623
age_l:race3Others	-0.31207	0.273339	0.253606
race1Black:male	0.020004	0.1691	0.905832
race2Hispanic:male	-0.35729	0.122736	0.003609
race3Others:male	-0.16571	0.177714	0.351112
race1Black:hfnc_or_niv	-0.12601	0.281766	0.654728
race2Hispanic:hfnc_or_niv	0.121979	0.201775	0.545495
race3Others:hfnc_or_niv	0.062851	0.309599	0.83913
race1Black:vent	-0.07718	0.230543	0.737807
race2Hispanic:vent	-0.34821	0.164334	0.034103
race3Others:vent	-0.22444	0.242187	0.354069
race1Black:creatinine_s	0.088356	0.082402	0.283732

© 2023 Fawzy A et al. *JAMA Network Open.*

race2Hispanic:creatinine_s	0.103258	0.069249	0.136043
race3Others:creatinine_s	0.087562	0.098698	0.375063
race1Black:bilitot_s	-0.00502	0.180268	0.977824
race2Hispanic:bilitot_s	-0.01136	0.126441	0.92847
race3Others:bilitot_s	0.250797	0.167025	0.134363
race1Black:hgb_s	0.077343	0.080394	0.336045
race2Hispanic:hgb_s	0.156007	0.055225	0.004742
race3Others:hgb_s	0.113104	0.077163	0.142757
race1Black:diabetes	-0.15199	0.203776	0.455756
race2Hispanic:diabetes	0.128217	0.145202	0.377245
race3Others:diabetes	-0.25707	0.20368	0.206937
race1Black:cci1	-0.29555	0.275147	0.282781
race2Hispanic:cci1	-0.28387	0.182825	0.120521
race3Others:cci1	0.035879	0.253776	0.887573
race1Black:cci2	0.117632	0.330089	0.721573
race2Hispanic:cci2	-0.30585	0.229625	0.182906
race3Others:cci2	0.15196	0.320844	0.635778
race1Black:smoking	0.246933	0.360171	0.492976
race2Hispanic:smoking	0.006723	0.290339	0.981528
race3Others:smoking	0.154906	0.436574	0.722729

© 2023 Fawzy A et al. *JAMA Network Open.*

eTable 4. Results of linear mixed models for the association of unrecognized need for COVID-19 therapy with time to COVID-19 therapy and readmissions

	**aHR**	**95% Confidence Interval**
Time to COVID-19 Therapy		
unrecognized	0.90	0.83-0.97
raceBlack	0.92	0.82-1.03
raceHispanic	1.13	1.02-1.25
raceOthers	1.13	0.99-1.30
age	1.00	1.00-1.00
Female	0.99	0.92-1.07
bmi	1.01	1.01-1.02
cci1	0.85	0.76-0.96
cci2	0.630	0.55-0.72
Readmission		
unrecognized	2.41	1.39-4.18
raceBlack	0.689003	0.34-1.38
raceHispanic	0.975716	0.54-1.75
raceOthers	1.245674	0.56-2.79
age	1.000213	0.98-1.02
Female	1.432148	0.89-2.31
who4	0.343735	0.12-0.95
who5	0.760906	0.39-1.50
who6	0.407724	0.21-0.78
bmi	1.008682	0.98-1.04
cci1	1.159821	0.50-2.66
cci2	1.887592	0.80-4.47

© 2023 Fawzy A et al. *JAMA Network Open.*
