# Supplementary material for: Clinical Outcomes Associated With Overestimation of Oxygen Saturation by Pulse Oximetry in Patients Hospitalized With COVID-19
Source: JAMA Netw Open. 2023 Aug 24;6(8):e2330856. doi: 10.1001/jamanetworkopen.2023.30856 (PMC10450566; doi:10.1001/jamanetworkopen.2023.30856)
Supplement: Supplement 2. — Data Sharing Statement [file jamanetwopen-e2330856-s002.pdf]

Data Sharing Statement

Fawzy. Clinical Outcomes Associated With Overestimation of Oxygen Saturation by Pulse Oximetry in Patients Hospitalized With COVID-19. *JAMA Netw Open*. Published August 24, 2023. doi:10.1001/jamanetworkopen.2023.30856

Data
Data available: No

Additional Information
Explanation for why data not available: The data that support the findings of this study are available from HCA Healthcare. Restrictions apply to the availability of these data, which were used under an interinstitutional agreement through the COVID-19 Consortium of HCA Healthcare and Academia for Research GEneration (CHARGE) for this study. Inquiries may be directed to co-author Kenneth Sands at kenneth.sands@hcahealthcare.com.
